# Counteracting conspiracy ideas as a measure of increasing propensity for COVID-19 vaccine uptake in Russian society

**DOI:** 10.7189/jogh.12.03013

**Published:** 2022-03-26

**Authors:** Dmitry V Boguslavsky, Konstantin S Sharov, Natalia P Sharova

**Affiliations:** Koltzov Institute of Developmental Biology of Russian Academy of Sciences, Moscow, Russia

## COVID-19-RELATED CONSPIRACY THEORIES AS A FACTOR OF VACCINE UPTAKE HESITANCY

Hardly any other disease or health care crisis of the past decades had led to a rise of conspiracy theories comparable to that of the COVID-19 pandemic, with many COVID-19-related theories being spread over social networks like an “infodemic”, ie, a separate pandemic of social moods accompanying the COVID-19 pandemic [1-6]. It was found in several countries that different SARS-CoV-2 vaccination campaigns suffered seriously due to the spread of conspiracy beliefs and related social resistance [7-10].

Some scientists, scholars, and noted political figures, including Sucharit Bhakdi [11,12], Marc Siegel [13], Slavoj Žižek [14], and Klaus Martin Schwab [15], believe that the growth of COVID-19-related conspiracy theories against COVID-19 vaccination may be accounted for by allegedly inconsistent public policy. However, most researchers note that the enormous surge of conspiracy ideas may be well explained by unprecedented levels of globalisation [16,17], digitisation [17], cultural unification [18], diminished levels of information quality/controllability in pandemic times [19], “bloggification” (the emergence of an almost endless amount of sources of information and centres of ideological influence on the Internet: personal blogs, independent media, independent health care experts, etc.) [20], and distrust towards the public policy [21]. Social media has been a useful vehicle for the instantaneous spread of conspiracy ideas about COVID-19 vaccination across huge audiences, a phenomenon that was not a feature of former epidemics, pandemics, or health care crises.

Thus far, there were contradictory suggestions in Russian media on the role of conspiracy ideas in impeding the Russian vaccination campaign. The RBC media agency assumed that conspiracy theories played a significant role in it [22], while Moscow24 declined it [23]. We believe that understanding if the relatively low vaccination rates in Russia (69 539 763 persons as of January 25, 2022, or 47.7% [24]) are related to the support of conspiracy theories by the general population is a crucial factor for the total success of the Russian vaccination campaign.

**Figure:**
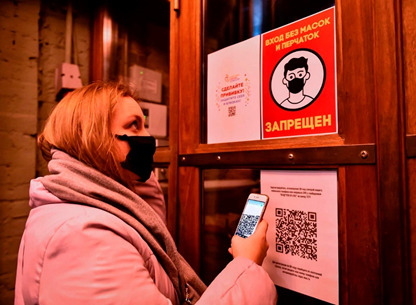
Photo: one of the most important factors in the spread of COVID-19-related conspiracy beliefs in Russia is the planned nationwide system of vaccination certification/verification based on QR codes. In the photo, the announcements on an entrance door read (in Russian): “Vaccinate! Protect yourself and your relations” (top left, with a small QR sign); “Entrance without masks and gloves is prohibited” (top right, red); and “Register by scanning the QR code with your smartphone camera or send an SMS “VHOD*078-875-253” to the number 7377. For the next QR code-based registration, click on the web link received, then input your mobile phone number and register via the mos.ru system” (bottom, with a QR code sign). For an ordinary person, that sequence of actions (vaccination verification before the entry to the premises) would take approximately 20-30 minutes at least. An inflexible technocratic approach may plunge a lot of people in Russia into supporting anti-vaccination conspiracy beliefs. © Chapaevsk Live https://smartik.ru/chapaevsk/post/141875329.

## INFLUENCE OF CONSPIRACY BELIEFS ON THE RUSSIAN VACCINATION CAMPAIGN

Through autumn and winter 2021, we carried out a biosocial survey via the Russian social network VKontakte (“In Contact”) to assess the role of conspiracy ideas in hampering SARS-CoV-2 vaccination. A thorough description of the surveying methodology may be found in our study devoted to another research goal [25]. There were 5822 respondents in total.

Around 54% of the individuals cautious about vaccination were found to support one or more conspiracy beliefs, or around one-fifth of the total sample set. Their geographical distribution is more or less even across different regions of Russia (all 85 Russian regions were represented in the survey but to a different degree) and there is no significant correlation between their place of residence and the local vaccination rate (Pearson correlation coefficient C = 0.1583 at *P* ≤ 0.3042). That makes Russia a country with one of the highest levels of support for COVID-19-related conspiracy beliefs [26-29]. Russian conspiracists are mainly people in their twenties (mean age 26.2 ± 6.6 years old, 95% CI, *P* = 0.05).

**Figure 1:**
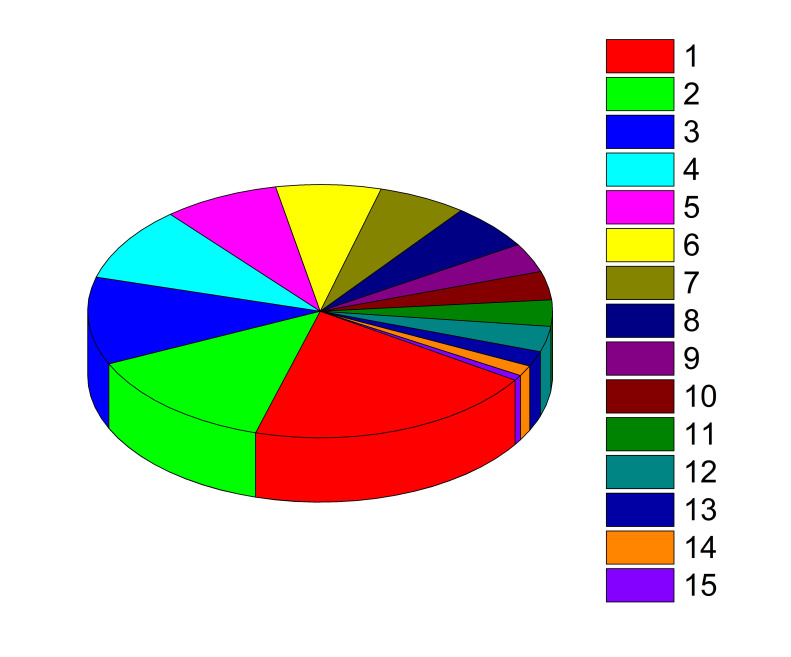
Percentage of support for different conspiracy theories in the group of “believers” in conspiracy ideas (1323 persons).

The content analysis allowed us to find basic characteristics of pandemic-related conspiracy beliefs and their connection with persistent media narratives, including social media. They are summarised in **Table 1****.** **Figure 1** demonstrates support of different conspiracist ideas. One can see that some conspiracy theories are universal, and others are specific to Russia.

**Table 1:** COVID-19-related media narratives, COVID-19-related conspiracy theories and corresponding mistrustful health behavioural responses that may be found in Russia*

No.*	Media narrative	Conspiracy belief	Main groups of the population that share/support the belief	Potential health behavioural response
1	Media boom about the forthcoming nationwide vaccination certification/verification system based on QR codes	Transforming Russia to a “digital concentration camp”	Different groups of the population	Active anti-vaccination behaviour
2	Media threats against those who express their doubts regarding the course, effectiveness, or outcome of vaccination against COVID-19, calling all of them “anti-vaxxers”	Forced transformation of people to “robots” without free will, by means of some electronic nanochip implanted through massive compulsory vaccination, including remote control, eg, the one ensured by 5G communication systems	Fundamentalist religious sect representatives	Active anti-vaccination behaviour
3	Media stressing advantages of online distance learning, transitioning to epidemiologically safe e-learning systems; the necessity of closure of schools and universities for an indefinite period	The Russian government uses the pandemic situation to lower the educational level of the population and, therefore, the level of critical reasoning. Thus, a caste of new slaves will be created to serve the needs of the future “Gold Billion”	School teachers, academia, researchers, people of the arts	Boycotting the online educational systems, ignoring possibilities of telemedicine systems
4	Official informational resources on COVID-19 statistics were dim and insufficient in quality and amount. Mostly, no important data about the pandemic were provided for a long time (up to the end of 2020)	SARS-CoV-2 does not exist. It is a hoax	Different groups of the population	Ignoring any medical advice, public health interventions and/or symptoms
5	Media emphasising that closing Christian temples, Muslim mosques and other sacred worshipping places without closing grocery stores will stop the pandemic	The Russian government is controlled by upcoming anti-Christ forces and the Apocalyptic times are near	Religious people or their supporters	Ignoring any epidemiological risk
6	Threats of introducing new administrative and/or criminal offence laws against those who make “disinformation on COVID-19” publicly available. Financial penalties and other measures for violation	“Police state” dawn in Russia with a transition to total “digitisation” of people	Religious people or their supporters, political opposition	Counteracting government-backed health care measures
7	Media psychosis on official TV channels about the doomed humanity. Searching for geopolitical enemies of Russia that may be more severely stricken with COVID-19, by pro-governmental media	The virus properties were invented by media and supra-governmental forces	People more accustomed to critical reasoning	Ignoring most medical advice, public health interventions and/or symptoms
8	Constant media talks about CCTV surveillance of almost every human and vehicle, financial penalties for disobedience	Russia is about to transform to Orwell’s 1984 society, a police state with total control of citizens and all personal freedoms cancelled	Working people	Intensified social communication with constant relocations, increased rates of droplet virus transmission
9	Media called SARS-CoV-2 “deadly” repeatedly without any necessary explanations of its “deadliness”	The Russian government is aware of the artificial origin of the coronavirus (from a test tube)	Scientific researchers, academia, university faculty	Avoiding vaccination
10	Media communication about salvation in the compulsory wearing of individual protecting units (masks and gloves) on the streets	This is a plot to enrich individual protective units makers (Russian oligarchs)	Aged persons, chronically ill persons	Ignoring and opposing wearing of individual protecting units, even in premises where they may be useful
11	Media communication about the need of switching off economies for long periods, without proving its necessity	COVID-19 has nothing to do with this. World governments want mass impoverishment of the majority of people, with enormous enrichment of the world elites from behind the veil	Self-employed, businesspersons, those who lost their businesses	Sabotaging any COVID-19 health care surveillance, prevention, and treatment measures
12	Multiple prolongations of quarantine and isolation measures, without assessing and proving their necessity to the general population	COVID-19 has nothing to do with this. World leaders have been maturing their plans for global totalitarianism for a long time, and now they agreed with each other to implement these plans simultaneously	Different groups of the population	Distrust in any COVID-19 numbers, suspecting lies everywhere, ignoring quarantine measures, deliberate appearance in public places when feeling ill
13	Strict media censorship. The firing of reporters and media commentators that disagree with the official governmental interpretation of COVID-19 information	All personal freedoms and/or opinions will soon be appropriated by the state	People of the arts, theatre, film circles, scientific researchers, religious people	Counteracting government-backed health care measures, ignoring any pieces of information about COVID-19
14	Requirements of self-isolation for aged people are much stricter than for younger persons	The Russian government wishes contractions of its budget loads by “abridging” of the aged population that must decrease due to side effects of isolation measures	Aged people	Intensified mobility of aged persons, increased risk for them to contract COVID-19 in grocery stores and during meetings with peers
15	Initial media support for chloroquine + azithromycin therapy (initial therapy for COVID-19 widely used till autumn 2020) as the only effective therapy for COVID-19 patients without distinguishing between personal conditions.	The Russian government is in conspiracy with world financial elites and transnational corporations wishes to curtail the Russian population by poisoning people with potentially dangerous chemicals	Hospital patients, those who had COVID-19 disease	Rejection to be treated with any medications

*The correspondence is made based on content analysis of the posts placed in Russian social network VKontakte by COVID-19-related conspiracists.

†The number corresponds with the rank of frequency of occurrence of a conspiracy belief in the Russian population (**Figure 1**).

We found that different conspiracy ideas were strongly interconnected. Table S in the **Online Supplementary Document** provides pairwise coefficients of correlation.

A considerable part of conspiracy beliefs leads to strong social resistance to COVID-19 vaccination campaigns in Russia. These beliefs are dangerous, as they may potentially undermine the effectiveness of a whole set of health care measures aimed at stopping the disease in the country, especially in the current situation of the fifth wave of the pandemic, caused primarily by the Omicron strain of SARS-CoV-2.

From **Table 1** and **Figure 1** (red segment), we see that a considerable part of conspiracy beliefs spread across the Russian society is concerned with the forthcoming introduction of the nationwide vaccination certification/verification system based on QR codes. Initially, the launch of the national QR code-based system was planned for February 2022. However, after many critical remarks from numerous health care experts, social organisations, religious organisations, non-governmental organisations, research institutions, and the general population, the parliamentary bill was returned to the government for improvement.

The objections to the QR code-based system form a separate important niche in the set of Russian conspiracy beliefs. They span twenty-two items and include moral, legal, economic, financial, social, infrastructural, political, philosophic, and aesthetic considerations.

## CONCLUSION

The main factors of relatively low vaccination rates in Russia are social, not medical. Many young people are more concerned with ensuring democracy, guaranteeing their rights of antagonism towards vaccination, and sustaining an open society than the national health or other medical considerations. A good approach may be demonstrating to them that their personal demands of securing their rights lead to violation of the rights of others, the rights of epidemiological safety, and diminishing COVID-19-related burdens.

While merely 16% of the sample set had doubts related to vaccine safety, 42% demonstrated at least a cautious attitude towards vaccination. Almost 23% of respondents supported one or more conspiracy ideas regarding the Russian COVID-19 vaccination campaign. These persistent beliefs in insidious social/political actors that allegedly use COVID-19 vaccination for achieving their pernicious goals are based upon psychological uncertainty and social fears associated with pandemic times. Even though many conspiracy ideas relate to vaccination only indirectly (**Table 1**), they do impede the Russian vaccination campaign, as our survey showed (strong interconnectedness of beliefs shown in Table S, **Online Supplementary Document**).

Therefore, we suggest the following measures for authorities that may increase the propensity of the Russian population to get vaccinated and dampen the fears that nourish conspiracist ideas:

- Increasing informational transparency and informational quality of media coverage of the Russian vaccination campaign;
- Encouraging participation of society in open debates about vaccination;
- Promoting information about vaccines in social networks, effectively elevating vaccine uptake;
- Supporting dialogue with religious and social organisations;
- Ensuring self-control, self-criticism, and feedback based on sentiments of the Russian society regarding vaccination – these sentiments can be detected and analysed in sociological surveys;
- Exchanging vaccines with other states, allowing foreign vaccines to the Russian market and promoting the Russian vaccines to foreign markets;
- Enhancing the quality of the vaccines used;
- Unbiased addressing the main conspiracist ideas in media;
- Avoiding disparaging and intimidating rhetoric against the opponents of vaccination and guaranteeing straightforwardness and goodwill to the general population.

## Additional material


Online Supplementary Document
