# Supplementary material for: Counteracting conspiracy ideas as a measure of increasing propensity for COVID-19 vaccine uptake in Russian society
Source: J Glob Health. 2022 Mar 26;2:03013. doi: 10.7189/jogh.12.03013 (PMC8942296; doi:10.7189/jogh.12.03013)
Supplement: Online Supplementary Document [file jogh-12-03013-s001.pdf]

Table S. Interconnectedness of different conspiracy theories. Pearson correlation coefficients C_{ab} are shown above the main grey diagonal (upper right corner of the Table) and corresponding significance levels P_{ab} below it (lower left corner). Calculation is done in Statistica 10 (StatSoft Inc., Tulsa, OK, USA).

**Theory**	**1**	**2**	**3**	**4**	**5**	**6**	**7**	**8**	**9**	**10**	**11**	**12**	**13**	**14**	**15**
**1**		0,9493	0,9269	0,9581	0,9406	0,9558	0,9511	0,9347	0,8756	0,9371	0,9324	0,8479	0,7572	0,8117	0,7793
**2**	$6{,}62\cdot10^{-8}$		0,95606	0,98781	0,9718	0,9939	0,9586	0,9550	0,9066	0,9579	0,9743	0,8729	0,7866	0,8389	0,8036
**3**	$6{,}79\cdot10^{-7}$	$2{,}67\cdot10^{-8}$		0,9820	0,8989	0,9667	0,9883	0,9925	0,9698	0,9965	0,9881	0,9458	0,8151	0,9059	0,8739
**4**	$1{,}97\cdot10^{-8}$	$6{,}93\cdot10^{-12}$	$8{,}58\cdot10^{-11}$		0,9551	0,9862	0,9747	0,9724	0,9235	0,9792	0,9818	0,8903	0,7708	0,8454	0,8091
**5**	$1{,}82\cdot10^{-7}$	$1{,}52\cdot10^{-9}$	$5{,}22\cdot10^{-6}$	$3{,}03\cdot10^{-8}$		0,9535	0,9007	0,8994	0,8238	0,8982	0,9052	0,7809	0,6665	0,7292	0,6968
**6**	$2{,}77\cdot10^{-8}$	$7{,}54\cdot10^{-14}$	$4{,}48\cdot10^{-9}$	$1{,}48\cdot10^{-11}$	$3{,}81\cdot10^{-8}$		0,9779	0,9733	0,9370	0,9739	0,9866	0,9122	0,8401	0,8836	0,8531
**7**	$5{,}2\cdot10^{-8}$	$1{,}81\cdot10^{-8}$	$5{,}23\cdot10^{-12}$	$7{,}72\cdot10^{-10}$	$4{,}66\cdot10^{-6}$	$3{,}14\cdot10^{-10}$		0,9961	0,9742	0,9942	0,9901	0,9562	0,8617	0,9262	0,8943
**8**	$3{,}31\cdot10^{-7}$	$3{,}07\cdot10^{-8}$	$2{,}84\cdot10^{-13}$	$1{,}35\cdot10^{-9}$	$5{,}04\cdot10^{-6}$	$1{,}08\cdot10^{-9}$	$4{,}00\cdot10^{-15}$		0,9828	0,9974	0,9883	0,9640	0,8522	0,9314	0,9032
**9**	$1{,}89\cdot10^{-5}$	$3{,}17\cdot10^{-6}$	$2{,}38\cdot10^{-9}$	$9{,}01\cdot10^{-7}$	$1{,}59\cdot10^{-4}$	$2{,}63\cdot10^{-7}$	$8{,}74\cdot10^{-10}$	$6{,}24\cdot10^{-11}$		0,9795	0,9700	0,9952	0,9087	0,97845	0,9611
**10**	$2{,}61\cdot10^{-7}$	$2{,}02\cdot10^{-8}$	$2{,}00\cdot10^{-15}$	$2{,}18\cdot10^{-10}$	$5{,}42\cdot10^{-6}$	$9{,}41\cdot10^{-10}$	$5{,}20\cdot10^{-14}$	$4{,}44\cdot10^{-16}$	$1{,}99\cdot10^{-10}$		0,9921	0,9594	0,8447	0,9259	0,8970
**11**	$4{,}14\cdot10^{-7}$	$8{,}52\cdot10^{-10}$	$5{,}68\cdot10^{-12}$	$9{,}14\cdot10^{-11}$	$3{,}47\cdot10^{-6}$	$1{,}23\cdot10^{-11}$	$1{,}70\cdot10^{-12}$	$5{,}18\cdot10^{-12}$	$2{,}26\cdot10^{-9}$	$4{,}02\cdot10^{-13}$		0,9496	0,8658	0,9238	0,8915
**12**	$6{,}51\cdot10^{-5}$	$2{,}16\cdot10^{-5}$	$1{,}01\cdot10^{-7}$	$8{,}66\cdot10^{-6}$	$5{,}89\cdot10^{-4}$	$2{,}16\cdot10^{-6}$	$2{,}59\cdot10^{-8}$	$7{,}40\cdot10^{-9}$	$1{,}58\cdot10^{-14}$	$1{,}60\cdot10^{-8}$	$6{,}36\cdot10^{-8}$		0,9346	0,9896	0,9790
**13**	0,00108	$5{,}03\cdot10^{-4}$	$2{,}1\cdot10^{-4}$	$7{,}69\cdot10^{-4}$	0,00665	$8{,}85\cdot10^{-5}$	$3{,}63\cdot10^{-5}$	$5{,}47\cdot10^{-5}$	$2{,}74\cdot10^{-6}$	$7{,}41\cdot10^{-5}$	$3{,}02\cdot10^{-5}$	$3{,}35\cdot10^{-7}$		0,9632	0,9621
**14**	$2{,}38\cdot10^{-4}$	$9{,}24\cdot10^{-5}$	$3{,}33\cdot10^{-6}$	$7{,}20\cdot10^{-5}$	0,00203	$1{,}25\cdot10^{-5}$	$7{,}18\cdot10^{-7}$	$4{,}51\cdot10^{-7}$	$2{,}74\cdot10^{-10}$	$7{,}40\cdot10^{-7}$	$8{,}77\cdot10^{-7}$	$2{,}37\cdot10^{-12}$	$8{,}50\cdot10^{-9}$		0,9932
**15**	$6{,}15\cdot10^{-4}$	$3{,}07\cdot10^{-5}$	$2{,}06\cdot10^{-5}$	$2{,}58\cdot10^{-4}$	0,00389	$5{,}26\cdot10^{-5}$	$6{,}86\cdot10^{-6}$	$3{,}95\cdot10^{-5}$	$1{,}21\cdot10^{-5}$	$5{,}84\cdot10^{-5}$	$8{,}10\cdot10^{-5}$	$1{,}87\cdot10^{-10}$	$1{,}03\cdot10^{-8}$	$1{,}44\cdot10^{-13}$	
